# Impulsiveness in Reactive Dieters: Evidence From Delay Discounting in Orthodontic Patients

**DOI:** 10.3389/fnhum.2018.00347

**Published:** 2018-08-31

**Authors:** Wu Zhang, Chunmiao Mai, Hongmin Chen, Huijun Zhang

**Affiliations:** ^1^The First Affiliated Hospital of Jinan University, Jinan University, Guangzhou, China; ^2^School of Stomatology of Jinan University, Jinan University, Guangzhou, China; ^3^School of Management, Guangdong University of Technology, Guangzhou, China; ^4^Center for Studies of Psychological Application, Guangdong Provincial Key Laboratory of Mental Health and Cognitive Science, School of Psychology, South China Normal University, Guangzhou, China; ^5^Mental Quality Education Center, Beijing Technology and Business University, Beijing, China

**Keywords:** orthodontic patients, reactive dieters, impulsiveness, intertemporal choice, subjective appetite

## Abstract

**Introduction**: Researchers have made efforts to distinguish the behavioral differences and underlying mechanisms that explain the various possible outcomes of dieting (success, failure and relapse). Although extensive research has demonstrated that eating behavior and individual impulsiveness are closely related to subjective appetite and decision making, very few studies have investigated how subjective and appetite impulsiveness is affected by reactive dieting.

**Methods**: In the present study, we utilized the power of food scale (PFS) and the intertemporal choice task and to examine subjective appetite and impulsivity of decision making in orthodontic patients. As a result of their orthodontic devices and the subsequent pain and discomfort caused by eating, these patients become reactive dieters. In order to explore the dynamic influence of orthodontic treatment on appetite and impulsiveness, we collected data for both patients and control participants across three testing sections. We also computed a regression model for further exploration in explaining how potential factors contributed to different choices.

**Results**: We found that the orthodontic group scored significantly lower in PFS than the control group, which indicated a suppression in appetite. Besides, reward and waiting time were significant factors in computational perspective. Moreover, although patients showed a bias in choosing smaller, immediate reward options, they exhibited a decrease in the delay discounting rate as treatment progressed. These findings confirm that subjective appetite and impulsiveness were inhibited due to reactive dieting.

## Introduction

People proactively control their diet for a variety of reasons, such as weight control, keeping fit, reducing blood glucose and lipids, religious fasting. During the dieting process, one has to overcome food temptation or even ignore nutritional needs to achieve a healthy balance between subjective desires and objective goals. For dieting to be successful, previous eating habits need to be changed by inhibiting dietary needs. Consequently, the inhibition capacity of each individual plays a key role in whether they will succeed in dieting or not.

Eating habits are also closely related to individual impulsiveness. Generally, impulsive individuals find it difficult to manage their diet and develop good eating habits. This phenomenon has been observed in a variety of diverse groups (Nederkoorn et al., 2006a,b; van den Berg et al., 2011; Weafer and Fillmore, 2012). More specifically it has been found that impulsiveness is closely related to inhibition capacity. Extensive research had demonstrated that bingeing individuals show lower inhibition capacity in comparison to healthy controls towards food stimuli in response inhibition tasks (Volkow and Wise, 2005; Grosshans et al., 2011; Lyu et al., 2016). Personal body mass index (BMI) has been seen to be positively associated with reaction time in a stop-signal task among impulsive individuals (Mühlberg et al., 2016), demonstrating that people with high trait impulsivity and poor control in weight have the difficulty in inhibiting responses. Individuals with relatively low inhibition capacity have been shown to consume much more high-calorie foods than those with a higher inhibition capacity (Houben, 2011; Houben and Jansen, 2011). In a recent study, dieters passively viewed food cues while activity in the inferior frontal gyrus, a brain region associated with inhibitory control, was measured (Lopez et al., 2016). Results showed reduced activation in this region in dieters with high desire to food compared to the dieters with low desire. This suggests that more cognitive resources were required to inhibit food stimuli-related impulsivity in order to balance the cognitive conflict between food temptation and dietary needs (Keller and Hartmann, 2016). These results suggest that top-down regulation of appetite plays a key role in determining whether dieters were able to diet successfully. This is consistent with the notion that regulation ability determines whether people can control eating behavior effectively (Papies et al., 2008; Werthmann et al., 2011).

Therefore, the training to improve regulation ability has been used as an approach to inhibit appetite for specific foods in individuals (Houben and Jansen, 2011; Houben, 2011; Veling et al., 2011a; Forman et al., 2016; Adams et al., 2017). This training was particularly effective for long-term dieters (Veling et al., 2011b). Results showed that foods related to nogo-signals were rated lowly while foods related to go-signals were rated more highly.

The above studies demonstrate that appetite control and the ability to behaviorally inhibit food-related stimuli are closely related. In addition to this line of research, the relationship between appetite and inhibition within the context of decision making has also been examined (Bartholdy et al., 2016). For instance, a study has found that the ability of overweight women to choose to delay gratification is positively correlated to their ability to inhibit food. Indeed, this correlation was more significant when they were shown food-related stimuli compared to when they were shown non-food stimuli, indicating that impulsiveness on decision making tasks can be influenced by inhibition capacity of food-related stimuli (Yeomans and Brace, 2015). Overall, these results offer some suggestion that eating and appetite do not only involve behavioral inhibition of food stimuli, but are also likely to engage more complex processes of impulsivity inhibition relating to goal-based decision making.

However, the aforementioned studies only focused on the participants with obesity or eating disorder, and thereafter have difficulty in distinguishing the effect of dietary habits from the effect of inherent factors, e.g., metabolic capability. In addition, the motivation of proactive dieter who changes his or her eating habits intentionally is associated with inhibiting behavior. Given the scarcity of relevant studies, we aimed to explore more fully the question of whether dietary habits or appetite influence impulsiveness in normal individuals.

Orthodontic treatment has a great impact on both the eating habits and appetite of patients especially in the early stage of the procedure. During this stage, arch wire attachments are used to apply direct force to irregular teeth. This inflicts pain and discomfort on patients, which in turn causes dietary restriction. This leads to an alteration in the eating habits of orthodontic patients such that they become more restricted. Thus, the application of orthodontic devices triggers a conflict between the desire to eat food and the oral pain and discomfort that they experience as a consequence of trying to satisfy this desire. In this way it is similar to individuals who proactively overcome food temptation in order to lose weight. That is, orthodontic patients have to overcome eating difficulties and various forms of discomfort caused by orthodontic treatment in the short term in order to achieve future improvement in oral aesthetics and functionality. But they have no specific motivation on diet. Given the notion that orthodontic devices cause patients to modulate their diet in a reactive manner, we can argue that changes in eating habits may affect their ability to balance short-term difficulties with long-term improvements. Through studies of orthodontic patients, it will be possible to develop a deeper understanding about how individual eating habits and appetite influence impulsivity in decision making.

Such a conflict between immediate discomfort and future interests lends itself to intertemporal choice (Frederick et al., 2002). This refers to choices in which individuals must make a tradeoff between costs and benefits occurring at different times (Liang and Liu, 2011). The classic example of such a tradeoff involves smaller sooner (SS) rewards and larger longer (LL) rewards. From the perspective of a rational economist, people should choose larger delayed rewards to maximize their interest. However, people are generally biased towards more immediate rewards. This is especially true for individuals who are highly impulsive (Thaler, 1981; O’Donoghue and Rabin, 1999). Mazur (1984) proposed that people tend to discount the subjective value of money as a function of delays in time. He explained this choice pattern mathematically using a hyperbolic function: *SV = R/(1 + kT)*, where *SV* represents the subjective value of the delayed reward *R* associated with waiting time *T*, and *k* was the delay discounting rate. The first goal of this study was to explore whether appetitive changes brought about by orthodontic devices impact impulsivity in decision making during treatment. Impulsivity in this case refers to an increased preference for immediate rewards. More specifically this can be described as an increase in the delay discounting rate.

The classic intertemporal choice paradigm modulates two dimensions, available reward and waiting time. Traditionally a bias for immediate rewards has been explained as the result of subjective devaluation as a function of delayed time, as described by the hyperbolic discounting model (Mazur, 1984; Laibson, 1997). But other studies have put forward different perspectives. Hariri et al. (2006) found that high discounters exhibited greater ventral striatal activation in comparison to low discounters when they were faced with immediate rewards, demonstrating that high discounters overestimate immediate rewards rather than underestimate future rewards. Another ERP study has drawn similar conclusions that high discounters overvalue immediate rewards (Cherniawsky and Holroyd, 2013). However, some researchers consider that the bias to immediate rewards in high discounters is generated by amplified subjective temporal perception (Zauberman et al., 2009). Thus, the second goal of this study was to examine the delay discounting rate of orthodontic patients in more detail by determining the influence of appetitive changes on both the reward sensitivity and time sensitivity during intertemporal choice.

In this study, we utilized the power of food scale (PFS; Cappelleri et al., 2009) to evaluate subjective appetite and an intertemporal choice task to explore individual impulsiveness on decision making. We measured the performance of a group of orthodontic patients on these two tasks and compared it with a group of normal controls. In orthodontics, the treatment process is divided into various temporal periods (Brown and Moerenhout, 1991): early treatment (2 weeks after applying devices), middle adaptation (4 weeks later), and subsequent adaptation period (4 months later). In order to explore the dynamic effects of eating habits and appetite on impulsivity in decision making, we tracked behavior changes across three distinct sections: the first, before treatment; the second, early treatment; and the third, adaptation to treatment. Previous studies have observed that inhibition training in behavioral responding to food stimuli can lead to a subsequent devaluation in the attractiveness of those food items. Given this finding, we hypothesized that a reduction in appetite caused by orthodontic-related difficulties in eating would lead to a reduction in impulsive responding of patients. More specifically, patients would choose less SS options in the intertemporal task and show a related reduction in their delay discounting rate after the application of an orthodontic device. Meanwhile, the orthodontic devices led to restraint on patients’ daily diet instead of their monetary rewards. Therefore, we predicted that the time sensitivity of patients, rather than reward sensitivity, would change significantly as a result of orthodontic treatment. This prediction is driven by the assumption that, during treatment, patients would have had to overcome conflicts between long time-consuming treatment and urgent desires of improving appearance. A consequently weakened sensitivity to time as well an underestimation of subjective time perception would bring about a decrease in choices for SS options.

## Materials and Methods

### Participants

The orthodontic patients were recruited from the dental department of a public hospital and were devoted to the treatment of orthodontics. They had never received orthodontic treatment before our recruitment. Thirty-three of them finished all measures in three test sections. Participants who performed absolute choice preference in the task were excluded. After exclusion, the orthodontics group consisted of 30 patients who would undergo orthodontic treatment (11 males, 19 females; mean age = 23.40, SD = 5.78; education = 14.00, SD = 1.92). The control group consisted of 31 adults (7 males, 24 females; mean age = 22.42, SD = 2.29; education = 15.13, SD = 1.80). They were recruited from local community and campus. Groups were matched on sex (*χ^2^* = 1.45, *p* = 0.23) and age (*t*_(59)_ = 0.88, *p* = 0.38). But the orthodontics group had shorter education years than the control group (*t*_(59)_ = −2.37, *p* = 0.02). All participants were right-handed, had normal or corrected-to-normal vision, were not color-blind, had no psychiatric illness and had not experienced dieting or suffered from eating disorders.

All orthodontic patients wore Self-brackets (Demon Q) and Demon CuNiTi Round 0.014 from KaVo-Sybron Dental Co., Ltd (Shanghai).

This study was carried out in accordance with the recommendations of Ethics Guideline, the Human Research Ethics Committee for Non-Clinical Faculties in South China Normal University. The protocol was approved by the Human Research Ethics Committee for Non-Clinical Faculties in South China Normal University. All subjects gave written informed consent in accordance with the Declaration of Helsinki. They could gain 50 RMB as reward after finishing all the three tests.

### Measurement Scale

The PFS was developed to assess the psychological impact of food and as a measure of subjective appetite for palatable food (Cappelleri et al., 2009). It has since been revised into an Asian version (Yoshikawa et al., 2012). The scale consists of 15 items, involving three levels of food proximity: food available indicates the degree of the desire to eat when palatable food is available in the environment but not physically present, food present indicates the degree of the desire when palatable food is present at hand, food tasted indicates the degree of the desire when we taste palatable food but not consume it (Cappelleri et al., 2009). Each item is rated from one to five points. Higher total scores indicate a greater influence of palatable food in the three contexts with different food proximity, referred to participant’s decreased ability to resist food temptation and increasing odds of being obese.

### Task

**Figure 1:**
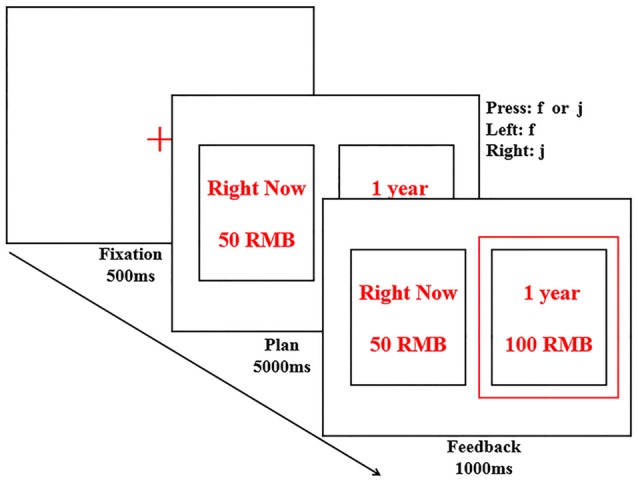
Illustration of the procedure in the intertemporal choice task. Subjects were asked to choose one of the options by pressing “f” or “j” key and shown feedback in red frame. In this trial, the participant chose an option with Δ Time of 1 year and Δ Reward of 50 yuan. In the actual experiment, options were presented in Chinese.

The intertemporal choice task required participants to select one of two monetary reward choices. One choice represented an immediate but smaller reward, the other one represented a larger but delayed reward. Participants were instructed that aim of the task was to assess how people make decisions about different monetary rewards. Each trial consisted of one option with a relatively smaller monetary reward (5, 10, 20, 30, 40, 50, 60, 70, 80, 90 yuan) obtainable right now (SS option), and one option with a relatively larger monetary reward (100 yuan) obtainable after a longer delay (LL option). Participants were asked to choose by pressing a key corresponding to the laterality of their preferred option I left or right). Reward sensitivity was calculated as the difference between every combination of LL and SS presented during the task (Δ Reward: 10, 20, 30, 40, 50, 60, 70, 80, 90, 95 yuan). Waiting time was calculated as the difference between the waiting time associated with LL and SS options (Δ Time: 1 day, 1 week, 1 month, 6 months, 1 year and 2 years). Each trial ran as follows: a fixation point (“+”) appeared in the screen center for 1,000 ms. Then reward options were presented for 5,000 ms or disappeared once an option had been selected. Following this, feedback on the participant’s choices was presented for 1,000 ms (see Figure 1). Pairs of stimuli were displayed randomly and counterbalanced across the left and right side of the screen. Each amount (from ¥5 to ¥ 95) and delay duration were repeated twice (one time per left and right side). The computerized task consisted of 120 trials.

### Procedure

All participants were required to participant in the behavioral tasks and fill all measurement scales involved in three separate sections. Experimental tasks and conditions were maintained the same for each section. The orthodontics group participated in the first section before they adopted the orthodontic treatment. This represented a baseline measure. The second section was conducted 2 weeks after the orthodontic devices have been put in place. This section aimed to probe how the orthodontic treatment had affected patient performance. The third and last section was conducted 6 weeks after the treatment. This represented the adaption phase for orthodontic patients at which point, according to customary orthodontic wisdom, patients will have gotten used to the treatment. Control participants completed all three sections in accordance with the time intervals of the patient group.

All data were collected in the behavioral lab affiliated with South China Normal University. Once participants arrived at the lab, they were given instructions about what was required from them during the experiment. All stimuli were presented on a 14-inch computer screen which was positioned 23 inches away from participant. Each experimental section consisted of two parts: the PFS scale and the intertemporal choice task. Sufficient practice was provided prior to the main tasks to ensure that participants were familiar with what was required of them. The whole procedure lasted 20 min and included breaks.

### Statistical Analysis

The data of subjects who performed absolute choice preference (always chose most of the SS or LL in three tasks) were eliminated from further analysis. Three patients who always chose SS options in all the three tests and two controls who always selected LL options were excluded. Reaction trials too fast (RT <500 ms) or too slow (RT >4,000 ms) were further eliminated. Over the three sections, 1%, 1% and 4% of trials in the orthodontics group respectively were removed as a consequence. The control group had 2%, 0.4% and 1% of trials removed.

This study aimed to explore the dynamic impact of orthodontic treatment on decision-making. We measured reward sensitivity (Δ Reward), time sensitivity (Δ Time), the percentage of SS choice, reaction time and delay discounting rate (*k* value) to quantify decision-making behavior. We conducted a 2 (group: orthodontics and controls) × 10 (Δ Reward: 10, 20, 30, 40, 50, 60, 70, 80, 90, 95 yuan) × 6 (Δ Time: 1 day, 1 week, 1 month, 6 months, 1 year and 2 years) × 3 (test time: first, second and third test) mixed design.

Data analyses were performed using SPSS 17.0 including *χ*^2^ test, independent *T*-test, repeated measures ANOVA, *post hoc* comparisons and logistics regression. Behavioral performances of the two groups were compared across the three research sections.

## Results

### Measurement Scale

**Figure 2:**
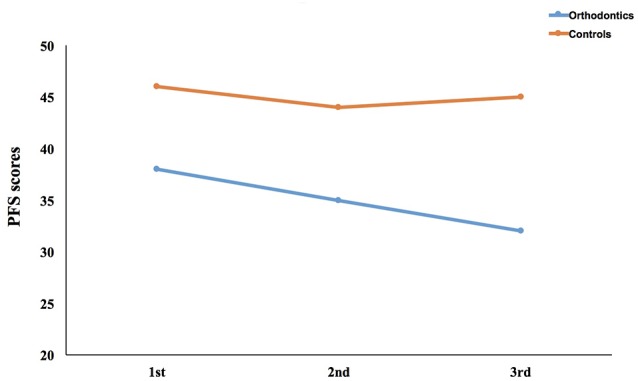
Results of power of food scale (PFS) across three sections. We calculated the PFS scores in all the three sections between the orthodontics group and the control group.

Scores for PFS of all participants were calculated (see Figure 2). We computed a mixed ANOVA with group as a between-subjects factor, test time as a within-subject factor and PFS scores as dependent variable. We found that the main effects for both group (*F*_(1,59)_ = 13.07, *p* = 0.001, $\eta_{\text{p}}^{2}$ = 0.18, *power* = 0.95) and time (*F*_(2,118)_ = 11.75, *p* < 0.001, $\eta_{\text{p}}^{2}$ = 0.17, *power* = 0.99) were significant. Multiple comparisons (LSD, Least Significant Difference; same as follows) showed differences between PFS scores taken from the first and second sections and between the first and third sections (*p* = 0.001 and *p* < 0.001 respectively). The interaction effect was also statistically significant (*F*_(2,118)_ = 7.13, *p* = 0.001, $\eta_{\text{p}}^{2}$ = 0.11, *power* = 0.93). A simple effects test showed that there were significant differences in PFS scores for the two groups across all three sections (all *p*s < 0.02). PFS scores for the orthodontics group were generally lower than those of the control group. More importantly, they showed a decrease over testing sections, which suggests that appetite declined with time.

### Reaction Time

We calculated a mixed ANOVA with reaction time as a dependent variable, group as a between-subjects factor (see Table 1). Choice (SS, LL) and test time were used as within-subject factors. The main effect of group was marginally significant (*F*_(1,59)_ = 3.42, *p* = 0.07, $\eta_{\text{p}}^{2}$ = 0.06, *power* = 0.44), while that of choice was not significant (*F*_(1,59)_ = 0.87, *p* = 0.36, $\eta_{\text{p}}^{2}$ = 0.02, *power* = 0.15). As the test time yielded a main effect (*F*_(2,118)_ = 42.29, *p* < 0.001, $\eta_{\text{p}}^{2}$ = 0.42, *power* = 1.00), *post hoc* tests were conducted and showed that the reaction time decreased significantly with testing sections (all* p*s < 0.001). Although the interaction effect between group and choice yielded a clearly significant difference (*F*_(1,59)_ = 6.94, *p* = 0.01, $\eta_{\text{p}}^{2}$ = 0.11, *power* = 0.74), none of the interaction effects reached the point of statistical significance (all* p*s > 0.6). A simple effects test indicated that participants from the orthodontics group were faster than those in the control group across all the three sections when selecting SS options (all *p*s < 0.05), however this was not evident when considering the LL option.

**Table 1:** Performance of reaction time.

RT (ms)		Orthodontics group		Controls group		Difference			
		*M*	*SD*	*M*	*SD*	*F*	Significance	$\eta_{\text{p}}^{2}$	Power
1st	SS	1491	425	1753	414	5.95	0.02	0.09	0.67
	LL	1632	448	1700	328	0.45	0.50	0.01	0.10
2nd	SS	1312	451	1546	390	4.70	0.03	0.07	0.57
	LL	1415	470	1511	334	0.86	0.36	0.01	0.15
3rd	SS	1211	409	1432	363	4.99	0.03	0.08	0.59
	LL	1285	451	1370	306	0.75	0.39	0.01	0.14

*Note: M indicates averaged reaction time of each group at each time point; SD indicates the standard deviation of reaction time of each group at each time point*.

### Choice Preference

To evaluate preference in choice between the two groups, we calculated a mixed ANOVA with percentage of SS options as a dependent variable (see Table 2). Only the main effect of group was evident (*F*_(1,59)_ = 20.70, *p* < 0.001, $\eta_{\text{p}}^{2}$ = 0.26, *power* = 0.99), revealing that participants from the orthodontics group showed a much stronger bias to SS option than those from the control group. Results from reaction time performance and choice preference confirm that orthodontics patients preferred to choose smaller and sooner rewards with greater impulsivity than control participants.

**Table 2:** Performance of choice preference.

	Group					
	Orthodontics group			Controls group		
Choice (%)	1st	2nd	3rd	1st	2nd	3rd
SS	0.71	0.64	0.65	0.44	0.43	0.43
SD	0.18	0.28	0.29	0.19	0.22	0.22

### Reward Sensitivity

Reward amount and waiting time were dominant factors affecting decision making. People would balance these two indices when considering SS and LL options. We quantified how monetary reward sensitivity and time sensitivity influenced decision behavior.

**Figure 3:**
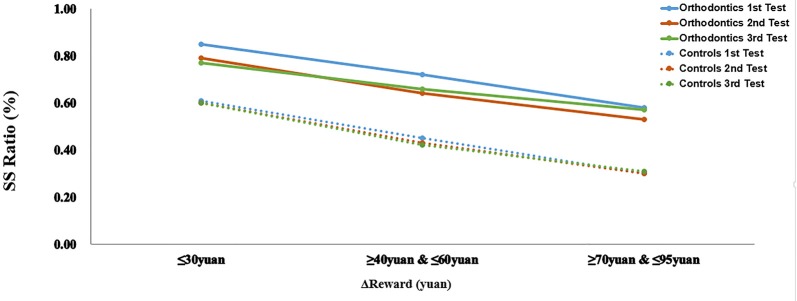
Performance as a function of Δ Reward. The percentage of smaller sooner (SS) choices are presented separately according to the increasing differences in reward magnitude between two groups across three testing sections.

The index of Δ Reward was grouped into three values: small Δ (10≤Δ≤30), medium Δ (40≤Δ≤60), large Δ (70≤Δ≤95). To examine the impact of reward sensitivity on choice performance between the two groups across different sections (see Figure 3), we conducted a mixed variance analysis using group as a between-subjects factor, Δ Reward and test time as within-subject factors and percentage of SS options chosen as the dependent variable. We found that the main effect of group was highly significant (*F*_(1,59)_ = 20.76, *p* < 0.001, $\eta_{\text{p}}^{2}$ = 0.26, *power* = 0.99), suggesting that participants from the orthodontics group performed with higher levels of impulsivity toward immediate rewards. The factor Δ Reward was also found to be significant (*F*_(2,118)_ = 134.88, *p* < 0.001, $\eta_{\text{p}}^{2}$ = 0.70, *power* = 1.00). Multiple comparisons revealed that participants chose a significantly higher percentage of SS options for small Δ compared to both medium Δ (*p* < 0.001) and large Δ (*p* < 0.001). They also chose a higher percentage of SS options for medium Δ compared to large Δ (*p* < 0.001). However, neither the main effect of test time, nor the interaction of two factors and three factors were significant. The two groups performed similarly with respect to their patterns of SS options as a function of Δ Reward.

### Time Sensitivity

**Figure 4:**
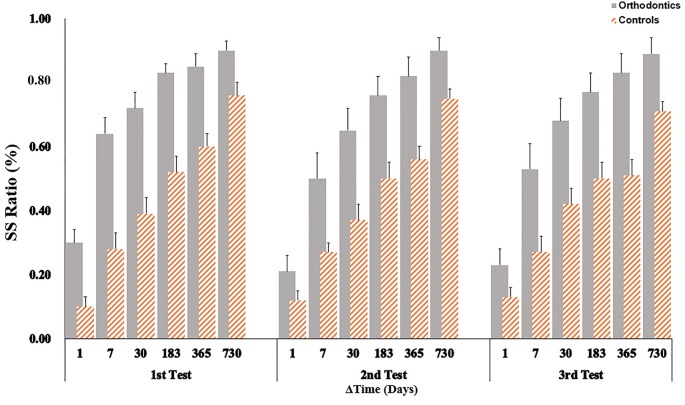
Performance as function of Δ Time. The percentage of SS choices were shown for six different time intervals. Orthodontic patients were biased to choose SS options in each waiting time condition for each section. Error bars showed one standard error.

In the following calculation, units of time were represented uniformly as days. The index of Δ Time was divided into six levels: 1 day, 7, 30, 183, 365 and 730 days. The performance of SS selection was calculated between the two groups across the three sections (see Figure 4). A mixed analysis of variance was fit to the data using percentage of SS options as the dependent variable, group was a between-subjects factor and Δ Time as a within-subject factor. We found that main effects for both groups (*F*_(1,59)_ = 20.94, *p* < 0.001, $\eta_{\text{p}}^{2}$ = 0.26, *power* = 0.99) and waiting time (*F*_(5,295)_ = 165.19, *p* < 0.001, $\eta_{\text{p}}^{2}$ = 0.74, *power* = 1.00] to be significantly different. Multiple comparisons showed that the two groups differed in every pairwise comparisons (*p* < 0.001). The interaction effect between groups and waiting time was clearly significant (*F*_(5,295)_ = 4.22, *p* = 0.001, $\eta_{\text{p}}^{2}$ = 0.07, *power* = 0.96) while all other interaction effects were not significant. A simple effects results showed that two groups differed in all Δ Time conditions (all *p*s < 0.01) in the first section. The similar patterns were observed in both the second and third test (all *p*s < 0.05) except in the condition of waiting for 1 day, in which participants did not exhibit different time sensitivity. These results confirm that participants from the orthodontics group showed a greater bias for more impatient choices. A covariate analysis that took education into account did not make a difference to this pattern of results.

### Binary Logistic Regression in Choice

We computed multivariate unconditional logistics regression to assess how reward and waiting time contributed to the subjects’ choices in trial-by-trial levels. The possible independent variables were considered as Δ Reward and Δ Time. They were z-scored normalized before regression. The dependent variable was the choice (SS = 1, LL = 0). We measured regression coefficients *β*, odds ratio, 95% confidence interval as well as significance (in the level of *p* < 0.05), using forward stepwise regression based on maximum likelihood estimation. The overall results were reported in the following table (see Table 3).

**Table 3:** Overall model of Δ Reward and Δ Time in choice.

	Regression coefficients β	*SD*	Wald χ^2^	Odds ratio (OR)	95% CI	Significance
ΔReward	−2.35	0.03	4979.39	0.10	0.09–0.10	<0.001
ΔTime	2.65	0.04	5025.24	14.18	13.17–15.25	<0.001
Constant	0.27	0.02	194.14	1.30		<0.001

Results showed that two factors were significant in explaining the choice (respectively, β_ΔReward_ = −2.35, OR_ΔReward_ = 0.10, *p*_ΔReward_ < 0.001; β_ΔTime_ = 2.65, OR_ΔTime_ = 14.18, *p*_ΔTime_ < 0.001). The constant was also significant in the model (*β* = 0.27, OR = 1.30, *p* < 0.001). These results confirmed the meaningful relation between reward, waiting time and choice.

To further estimate the contribution of Δ Reward and Δ Time between two group in each test, we calculated the Regression coefficients *β* values respectively (see Table 4). The independent variables were group, *β* type and test time while the dependent variable was coefficient *β* value. We measured a three-factor mixed analysis of variance. However, no significant effect was found in either main effect or interaction effect.

**Table 4:** Contribution of Δ Reward and Δ Time between two group.

Regression coefficients β		Orthodontics group		Controls group		Difference			
		*M*	*SD*	*M*	*SD*	*F*	Significance	$\eta_{\text{p}}^{2}$	Power
1st	ΔReward	−2.64	0.10	−2.95	0.10	1.03	0.31	0.02	0.17
	ΔTime	3.05	0.12	3.17	0.11	0.97	0.33	0.02	0.16
	Constant	1.34	0.06	−0.70	0.06	0.53	0.47	0.01	0.11
2nd	ΔReward	−2.32	0.09	−2.67	0.09	1.03	0.31	0.02	0.17
	ΔTime	2.98	0.11	2.88	0.09	0.97	0.33	0.02	0.16
	Constant	0.99	0.05	−0.60	0.05	1.97	0.17	0.03	0.28
3rd	ΔReward	−2.10	0.08	−2.60	0.09	1.03	0.31	0.02	0.16
	ΔTime	2.65	0.10	2.71	0.09	0.97	0.33	0.02	0.16
	Constant	1.04	0.05	−0.57	0.05	1.87	0.18	0.03	0.27

### Delay Discounting Function

To examine how subjects discounted rewards according to waiting time before and after orthodontic treatment, we calculated the discount factor for each testing section. Delayed discounts predicted the degree of how people devalued rewards over time. The discount rate is conventionally described through the hyperbolic function (Grossbard and Mazur, 1986; Mazur, 1987). In this study, the hyperbolic function was:

(1) SV=R/(1+kW)

In order to derive the discount rate of each participant (*k* parameter) when SS option was selected, Grecucci et al. (2014) had transformed equation 1 into formula as follows:

(2) k=(RLL−RSS)/[(RSS*WLL)−(RSS*WSS)]

In this formula, RLL represented the reward linked to LL option (RLL = 100), RSS represented the reward linked to SS option, WLL was the waiting time related to LL option and WSS was the waiting time related to SS option (WSS = 0). In this study, the formula could be simplified into: *k* = ΔReward/(RSS*Δ Time).

To compare the discounting rates between two groups over time, we used group and test time as independent variables with *k* value as the dependent variable and entered them into a mixed variance analysis (see Table 5). Results showed that the main effect of group was significant (*F*_(1,59)_ = 13.52, *p* = 0.001, $\eta_{\text{p}}^{2}$ = 0.19, *power* = 0.95) although test time was not significant (*F*_(2,118)_ = 0.87, *p* = 0.42, $\eta_{\text{p}}^{2}$ = 0.01, *power* = 0.20). A significant interaction effect was evident (*F*_(2,118)_ = 3.20, *p* = 0.04, $\eta_{\text{p}}^{2}$ = 0.05, *power* = 0.60), with a simple effects test indicating differences in pairwise comparisons (all *p*s < 0.05). The tendency to discount reward with time of orthodontics patients was greater than that of control participants across all three sections. *Post hoc* comparison of *k* value in the orthodontics group showed that there was an obvious difference between the first and second section (*p* = 0.04), while a significant difference was not observed between the second and third test (*p* = 0.84). This result demonstrated a remarkable decrease in the discounting rate of orthodontics in the second section.

**Table 5:** Discounting rate (*k* value) of both groups.

*k* value	Orthodontics group		Controls group		Difference			
	*M*	*SD*	*M*	*SD*	*F*	Significance	$\eta_{\text{p}}^{2}$	Power
1st Test	0.30	0.29	0.05	0.07	21.67	<0.001	0.27	0.996
2nd Test	0.20	0.26	0.07	0.15	5.82	0.019	0.09	0.660
3rd Test	0.23	0.29	0.08	0.19	5.27	0.025	0.08	0.617

Some research (Appelhans et al., 2012; Grecucci et al., 2014) had estimated “indifference points,” a balanced index between instant reward and delayed reward in the intertemporal task. It was supposed the point in which people selected 50% equally in SS option (or LL option). In this point, people valued equally in the two options. According to the equation transformation (Grecucci et al., 2014), the computation formula could be as follows:

(3) RSS/[1+(k*WSS)]=RLL/[1+(k*WLL)

These points were measured by converting the subjective values. In this study, the formula could be simplified into: WLL = (RLL − RSS)/(k*RSS). To devalue 100 RMB to 50 RMB in the 3rd section, the patients needed 4 days while the controls needed 13 days.

$$\begin{array}{c} {\text{WLL}}_{\text{patients}}=(100-50)/(0.23*50)=4\\ {\text{WLL}}_{\text{controls}}=(100-50)/(0.08*50)=13 \end{array}$$

**Figure 5:**
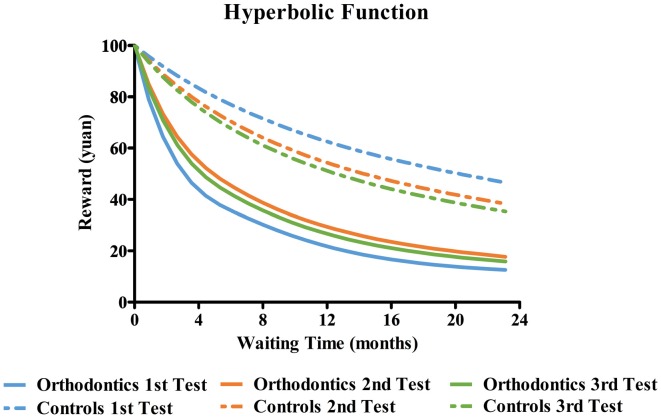
Waiting time and Hyperbolic Function. Fitted hyperbolic functions were plotted for a fixed reward of 100 yuan for both groups according to their *k* values across three sections. For the purposes of graphical presentation, the unit of waiting time was converted from “days” to “months”. As is illustrated, the orthodontic patients showed steeper discount function compared to control participants across all the three sections.

Hyperbolic function graphs were plotted in GraphPad Prism 5 in accordance with the degree of discount in two groups respectively across the three sections (as shown in Figure 5). These show that steepness of the discount curve was greater for the orthodontics group compared to the control group. We also calculated the area under the hyperbolic curve (AUC) for each participant in each section (see Table 6). We performed a mixed ANOVA on groups and test sections as independent variables and AUC as the dependent variable. The main effect of groups was markedly significant (*F*_(1,59)_ = 25.57, *p* < 0.001, $\eta_{\text{p}}^{2}$ = 0.30, *power* = 0.99) while that of test time was only marginally significant (*F*_(2,118)_ = 2.88, *p* = 0.06, $\eta_{\text{p}}^{2}$ = 0.05, *power* = 0.55). The interaction effect was also significant (*F*_(2,118)_ = 4.46, *p* = 0.01, $\eta_{\text{p}}^{2}$ = 0.07, *power* = 0.76). A further simple effects test revealed that AUC of orthodontics patients was clearly larger than controls across all three sections (all *p*s < 0.002). Consistent with patterns derived from *k* values, *post hoc* comparisons showed that the AUC increased significantly in the second test compared to the first test (*p* = 0.003) but it was similar to the third test (*p* = 0.65).

**Table 6:** Area behind the curve of two groups (Area).

Area	Orthodontics group		Controls group		Difference			
	*M*	*SD*	*M*	*SD*	*F*	Significance	$\eta_{\text{p}}^{2}$	Power
1st Test	1037	522	1887	464	45.26	<0.001	0.43	1.000
2nd Test	1329	627	1849	568	11.52	0.001	0.16	0.916
3rd Test	1301	640	1866	562	13.43	0.001	0.19	0.950

### Task Difficulty

To verify whether the task difficulty made a difference in the reaction time, we measure general linear regression to explore the effects of group and Δutility on reaction time. The choice utility was calculated according to the hyperbolic function: *SV = R/(1 + kT)*. The utility of SS choice was the SS reward while the utility of LL choice was the subjective value after devaluation of LL reward. The Δutility was the result of the difference between LL utility and SS utility.

The results showed that the regression model was significant (*F* = 217.28, *p* < 0.01). The factor of group was remarkable in the regression model (*t* = 85.53, *p* < 0.01) while the factor of Δutility was excluded to the model (*t* = 1.38, *p* = 0.17). 10% of the variation of the reaction time can be explained by changes in the group (corrected *R*^2^ = 0.01). These results revealed that reaction time was the result of the difference between two groups rather than the task difficulty.

### Individual Appetite and Delay Discounting

In order to explore the relationship between food sensitivity and impulsiveness of decision making in the intertemporal choice task, we computed Pearson correlation analyses between PFS scores (and three PFS subscale scores), as well as SS ratio, RTs of SS choice, and *k* value for the three sections. Results indicated that individual appetite and task performance were not significantly correlated (all *p*s > 0.1).

## Discussion

This study examined whether appetitive changes caused by difficulties in eating affected impulsivity in decision making. We did this by comparing performance in an intertemporal choice task before and after orthodontic treatment with a group who did not undergone this treatment. The results confirmed our hypothesis that the appetite of orthodontic patients would be suppressed after installation of orthodontic devices, and that there would be a subsequent decrease in impulsiveness as measured by the intertemporal choice task.

There has been a great deal of research on the relationship between individual impulsivity and dietary inhibition, as well as behavioral inhibition. Impulsivity and dietary inhibition may engage a common neural mechanism. For example, the intensity of activity in the brain region of known as the dorso-lateral prefrontal cortex (DLPFC) is generally regarded as an indicator of behavior inhibition capacity. It is closely related to biases in preference for SS options as well as the delay discounting rate in the intertemporal choice task (He et al., 2016; Wesley and Bickel, 2014), and is thought to predict the success rate of individuals in their endeavor to diet or inhibit eating (Weygandt et al., 2015). A serial study has found that the activation intensity of DLPFC during intertemporal choice was positively correlated with dietary inhibition (Dong et al., 2016). Spontaneous neuronal activity of DLPFC in the resting state has also been found to be negatively correlated with dietary inhibition (Dong et al., 2015). These results suggest that these two processes share common neural mechanism. As such, difficulties in eating or dietary inhibition are likely to affect individual impulsivity. Recent studies have illustrated that inhibition training of specific foods contributes to the suppression of food sensitivity and evaluation (Chen Z. et al., 2016; Chen et al., 2017). The researchers manipulated go-nogo training on participants by using food pictures as stimuli, as well as requiring them to evaluate the attractiveness of food pictures related to go-signal and nogo-signal before and after the training. This study supported the arguments that the evaluation to food was affected by individual’s inhibition tendency and that the effect of inhibition training might further affect subjective appetite. Our study was consistent with this notion. Thus, we interpret a persistent reduction in impulsiveness as indexed by the intertemporal choice task is a consequence of eating difficulties experienced by patients as a result of their orthodontics devices through the common neural circuit of behavioral inhibition and impulsivity (Chen S. et al., 2016). Our findings build on this evidence by suggesting that the impact of eating inhibition on behavior not only affect food-related behavior, but also can also be extended to general decision making tasks. In respect to decision making, some previous studies had computed regression analysis in intertemporal choice paradigm to explore the possible factors to the final choice (Appelhans et al., 2012; Grecucci et al., 2014). In our study, the results of regression model have demonstrated the significant contribution of reward factor and waiting time factor to the choice performance.

We found that patients’ subjective appetite for food changed dynamically as the course of the orthodontic treatment progressed. However, previous research in which proactive dieters were more strongly influenced by food after dieting over time suggests the opposite conclusion (Forman et al., 2007). We can put forward two possibilities for this discrepancy. One possibility is that self-report measures are unlikely to accurately reflect the subjective state of participants because of measurement bias and social commitment bias (Sayette et al., 2000). Usually obese individuals tend to underreport subjective appetite or hunger state because of social pressures or feelings of shame (Stunkard, 1959). Indeed, eating tasks have been found to be more effective than self-report measures for subjective appetite (Nijs et al., 2010). The second possibility might be that the orthodontics patients, as reactive dieters, inhibited appetite in order to eat less and to avoid pain and discomfort. This type of appetite inhibition can be described as a more reactive when compared to the more proactive response exhibited by individuals who choose to diet. Besides, this result didn’t support the assumption of the correlation between appetite and impulsiveness. Further studies would benefit from making use of more accurate and objective measures to explain subjective appetite.

In the present study, we observed a significant reduction in subjective appetite in reactive dieters. These were patients faced with pain and discomfort in eating due to the application of orthodontic devices. In addition, we found an associated decrease in impulsive monetary choices over time. According to the hyperbolic function, the smaller this delay discounting rate, the slower the individual discounted subjective value and the less impulsive they were in their decision making (Myerson et al., 2013). This finding demonstrated that the inhibition of eating behaviors regulated impulsivity inhibition in decision making, suggesting the presence of a common physiological mechanism.

It was revealed that the patients had shown a highly impulsive level at the beginning as compared to the controls. One possible explanation was due to their personality of impulsiveness. Wittmann and Paulus (2008) argued that individual differences in temporal perception were likely to affect the delay discounting rate in intertemporal choices to some extent. They observed that an overestimation of time led to an SS bias while underestimation of time led to more LL options. Orthodontic treatment is a medical procedure that carries patients with pain and eating difficulty. Most adult patients make a decision to undertake orthodontic treatment because they are unhappy with their facial appearance and the associated negative impact it has on their daily life. The strong conflict between eager to become good-looking and having to wait patiently are likely to drive them overestimate the time and result in a high impulsivity level.

## Limitation

A general limitation of the current study is the significantly higher impulsiveness level in patients as compared to controls in each test section, which make doubts in the generalization of findings about the inhibition effect of reactive dieting in impulsiveness. It is hard to explain this always highly impulsive level in patients at the behavioral perspective. Two groups were categorized based upon whether adopting orthodontic treatment rather than scale measurement. We didn’t control the initial level of impulsivity between two groups. However, it is notable that two groups were balanced in demographics and the grouping criteria was the orthodontic treatment. All the dynamic changes in patients were in result of the treatment effect, more specifically were due to reactive dieting. To control for potential confounding effects in impulsivity, it would be better to utilize the Barratt Impulsiveness Scale (Hollander et al., 1998) as the pre-inclusion standard. A longer tracking examination is helpful to reveal whether the impulsiveness level in patients will continuously decrease along with the treatment.

In respect of reward sensitivity and time sensitivity, they were not significantly affected by orthodontic treatment. The lack of such effects suggests that changes in task performance from patients across sections were related to the interaction of variation between reward magnitude and delay, rather than one specific factor.

An additional limitation is subjective appetite and impulsivity did not differ after a long-term adaptation of orthodontic treatment. Patients exhibited similar levels of food sensitivity and decision-making performance from one stage of treatment to the next. This implies that changes in behavioral and psychological aspects were not affected by the adaptation in 6 weeks after the orthodontic treatment. Whether there is a significant adaptive effect in subsequent treatment periods remains to be seen and would require further, longitudinal study. It would be beneficial to consider whether or not patients remained in a state of reactive dieting and experienced associated difficulties in eating when they are in the late stage of treatment.

## Conclusion

This novel study explored the subjective appetite and impulsiveness of decision making in reactive dieters. We found that both of these factors were affected by the restricted diet brought about by the application of orthodontic devices. Reactive dieting suppressed subjective appetite as well as individual impulsivity. This study adds to our understanding of how reactive dieting influences aspects of physiology and behavior.

## Author Contributions

WZ and HZ designed the study. CM and WZ collected research data. All four authors finished the manuscript.

## Conflict of Interest Statement

The authors declare that the research was conducted in the absence of any commercial or financial relationships that could be construed as a potential conflict of interest.
